# Neurometabolite alterations in traumatic brain injury and associations with chronic pain

**DOI:** 10.3389/fnins.2023.1125128

**Published:** 2023-02-23

**Authors:** Linda E. Robayo, Varan Govind, Teddy Salan, Nicholas P. Cherup, Sulaiman Sheriff, Andrew A. Maudsley, Eva Widerström-Noga

**Affiliations:** ^1^The Miami Project to Cure Paralysis, University of Miami, Miami, FL, United States; ^2^Neuroscience Graduate Program, University of Miami, Miami, FL, United States; ^3^Department of Radiology, University of Miami, Miami, FL, United States; ^4^Department of Neurological Surgery, University of Miami, Miami, FL, United States

**Keywords:** traumatic brain injury, chronic pain, *N*-acetylaspartate, brain metabolite, magnetic resonance spectroscopic imaging

## Abstract

Traumatic brain injury (TBI) can lead to a variety of comorbidities, including chronic pain. Although brain tissue metabolite alterations have been extensively examined in several chronic pain populations, it has received less attention in people with TBI. Thus, the primary aim of this study was to compare brain tissue metabolite levels in people with TBI and chronic pain (*n* = 16), TBI without chronic pain (*n* = 17), and pain-free healthy controls (*n* = 31). The metabolite data were obtained from participants using whole-brain proton magnetic resonance spectroscopic imaging (^1^H-MRSI) at 3 Tesla. The metabolite data included *N*-acetylaspartate, *myo*-inositol, total choline, glutamate plus glutamine, and total creatine. Associations between *N*-acetylaspartate levels and pain severity, neuropathic pain symptom severity, and psychological variables, including anxiety, depression, post-traumatic stress disorder (PTSD), and post-concussive symptoms, were also explored. Our results demonstrate *N*-acetylaspartate, *myo*-inositol, total choline, and total creatine alterations in pain-related brain regions such as the frontal region, cingulum, postcentral gyrus, and thalamus in individuals with TBI with and without chronic pain. Additionally, NAA levels in the left and right frontal lobe regions were positively correlated with post-concussive symptoms; and NAA levels within the left frontal region were also positively correlated with neuropathic pain symptom severity, depression, and PTSD symptoms in the TBI with chronic pain group. These results suggest that neuronal integrity or density in the prefrontal cortex, a critical region for nociception and pain modulation, is associated with the severity of neuropathic pain symptoms and psychological comorbidities following TBI. Our data suggest that a combination of neuronal loss or dysfunction and maladaptive neuroplasticity may contribute to the development of persistent pain following TBI, although no causal relationship can be determined based on these data.

## 1. Introduction

Survivors of traumatic brain injury (TBI) experience a wide range of physical and mental health problems, including sensorimotor, behavioral, and neuropsychological deficits (Ponsford et al., 2014; Bramlett and Dietrich, 2015; Gardner and Zafonte, 2016; Irvine and Clark, 2018; Ng and Lee, 2019). It is estimated that TBI contributed to approximately 224,000 annual hospitalizations and over 64,000 deaths in 2020 in the United States (Centers for Disease Control and Prevention [CDC], 2022). Domestic surveillance data reveal that between 3.2 and 5.3 million individuals are currently living with at least one TBI-related disability, which further emphasizes the burden of TBI in the US population (Centers for Disease Control and Prevention [CDC], 2015). Clinical studies suggest that many TBI-associated symptoms, including pain (Prins et al., 2013; Rabinowitz and Levin, 2014; Bramlett and Dietrich, 2015; Irvine and Clark, 2018; Ng and Lee, 2019; Pavlovic et al., 2019; Capizzi et al., 2020), persist over an extended period. Incidentally, more than 50% of individuals with TBI report chronic pain (Lahz and Bryant, 1996; Nampiaparampil, 2008). While pathophysiological mechanisms underlying the development of chronic pain after TBI are not entirely understood, those individuals reporting chronic pain frequently present with a combination of characteristics that resemble those of neuropathic pain conditions, including sensory alterations and psychological comorbidities (Ofek and Defrin, 2007; Widerström-Noga et al., 2016; Irvine and Clark, 2018; Khoury and Benavides, 2018; Bouferguène et al., 2019; Leung, 2020; Robayo et al., 2022). Thus, chronic neuropathic pain can be a consequence of trauma or diseases involving the central or peripheral nervous system, such as TBI (Ofek and Defrin, 2007; Scholz et al., 2019; Robayo et al., 2022).

Findings from preclinical TBI research can offer insights into the molecular and cellular basis for developing chronic neuropathic pain symptoms, including central and peripheral sensitization, prolonged disinhibition within thalamocortical circuits, and the derangement of glial cell functions (Ji et al., 2013; Groh et al., 2018; den Boer et al., 2019). In humans, significant mechanistic insights have been gained through pain phenotyping based on symptoms, signs, and biomarkers, including brain imaging. Indeed, neuroimaging studies have identified both functional and structural abnormalities within specific pain-related brain regions in individuals reporting neuropathic pain (Geha and Apkarian, 2005; Moisset and Bouhassira, 2007; Alshelh et al., 2016; Mills et al., 2018). Numerous brain regions, including the thalamus, anterior cingulate cortex (ACC), insula, prefrontal cortex (PFC), and somatosensory cortices (S1 and S2), are involved in processing sensory-discriminative (e.g., location and intensity), cognitive-evaluative, and affective-emotional aspects of pain (Bushnell et al., 2013; Kuner and Kuner, 2021; Mercer Lindsay et al., 2021). Each of these regions uniquely contributes to the overall modulation and resulting perceptual experience (Davis and Moayedi, 2013; Bastuji et al., 2016; Garcia-Larrea and Bastuji, 2018). The prefrontal cortex, has been implicated in attentional and evaluative processes of pain, including attention, anticipation, learning, and cognitive control (Bushnell et al., 2013; Tan and Kuner, 2021). Common findings reported in the literature include increased prefrontal cortex functional activation and decreased gray matter volume in individuals with chronic pain (Seminowicz and Moayedi, 2017). Interestingly, the activity of the prefrontal cortex can be modulated by practicing mindfulness and meditation (Allen et al., 2012). The insula has been implicated in coding pain intensity, whereby electrical stimulation of this structure appears to elicit pain, and lesions affecting the insula can lead to the development of neuropathic pain (Garcia-Larrea et al., 2010; Garcia-Larrea and Peyron, 2013). The insula receives input from the spinothalamic tract and has also been implicated in mediating complex cognitive processes, including emotional awareness (Craig, 2009; Starr et al., 2009). The anterior cingulate cortex (ACC) and cingulum are components of the limbic system, thought to be involved in the affective-emotional components of the pain experience (Bushnell et al., 2013; Kuner and Kuner, 2021). The ACC can modulate both sensory and affective aspects of pain via activation of various receptor systems, including μ-opioid and gamma-aminobutyric acid (GABA) receptors, and activation of the periaqueductal gray (PAG) (Fuchs et al., 2014; Benarroch, 2020). Preclinical studies show that the ACC can undergo profound functional and structural changes during acute and chronic pain (Bliss et al., 2016), and such changes have also been associated with depression in human patients (Holtzheimer et al., 2017). The postcentral gyrus or primary somatosensory cortex is commonly activated during induced pain and implicated in the sensory-discriminative (e.g., location and intensity) components of the pain experience (Apkarian et al., 2005; Kuner and Kuner, 2021; Tan and Kuner, 2021). Greater functional connectivity between the ACC and postcentral gyrus has been observed in adults with chronic pain (Youssef et al., 2019). Lastly, the thalamus is the primary relay station for sensory information, and it also communicates with the cortex (Apkarian et al., 2005; Kuner and Kuner, 2021). Furthermore, the thalamus is rich in opioid receptors and, therefore, may also be involved in pain modulation (Apkarian et al., 2005).

Chronic neuropathic pain has been associated with increased activation of the ACC, insula, PFC, S1, and S2 and decreased thalamic activity (Moisset and Bouhassira, 2007; Friebel et al., 2011; Lin, 2014), accompanied by an altered thalamocortical rhythm (Walton and Llinás, 2010; Alshelh et al., 2016). Similarly, studies evaluating structural abnormalities found lower volume in the thalamus, ACC, PFC, and anterior insula, and greater volume in the periaqueductal gray (PAG) and posterior insula in individuals with neuropathic pain compared to healthy controls (Pan et al., 2015; Henssen et al., 2019). These findings suggest that abnormalities in cortical and subcortical brain regions may alter the perception and modulation of pain signals (i.e., maladaptive pain processing) (Kuner and Flor, 2017) and result in chronic neuropathic pain.

Brain imaging modalities such as magnetic resonance imaging (MRI) and magnetic resonance spectroscopy (MRS) are often used to identify brain tissue and metabolic abnormalities associated with TBI (Brooks W. M. et al., 2001; Croall et al., 2015; Faghihi et al., 2017; Smith et al., 2019) and neuropathic pain conditions (Geha and Apkarian, 2005; Moisset and Bouhassira, 2007; Alshelh et al., 2016; Mills et al., 2018). Specifically, proton magnetic resonance spectroscopy imaging (^1^H-MRSI) is a non-invasive MRI technique that allows the acquisition of spectroscopic data for several brain metabolites, including N-acetyl aspartate (NAA- a proxy for neuronal density and viability), total choline (tCho – an indicator of cell membrane integrity), glutamate and glutamine (Glx – excitatory neurotransmitter and its precursor), total creatine (tCre – a biomarker of cellular energetics), and *myo*-inositol (*m*-Ins – an indicator for glial cell density and inflammation) (Miller et al., 1996; Govindaraju et al., 2000; Brooks W. M. et al., 2001; Bak et al., 2006; Moffett et al., 2007; Govind et al., 2010; Chang et al., 2013; Croall et al., 2015; Bartnik-Olson et al., 2019). While metabolite alterations across brain regions have been examined in multiple chronic pain conditions (Fukui et al., 2006; Chang et al., 2013; Widerström-Noga et al., 2013; Ito et al., 2017; Levins et al., 2019; Peek et al., 2020), including neuropathic pain (Chang et al., 2013; Widerström-Noga et al., 2015), only a few research studies have focused on those with TBI and comorbid pain (Widerström-Noga et al., 2016; Lin et al., 2022). Moreover, prior TBI studies have utilized single-voxel or multi-voxel-single slice or multi-slice MRS techniques (Babikian et al., 2010; Widerström-Noga et al., 2016) for the evaluation of brain metabolites within a region or multiple regions of interest (ROIs). However, these limited spatial coverage MRS techniques may not be sufficient to enclose injured brain regions spread across the brain; this might limit assessments of brain metabolites in cortical and subcortical brain regions responsible for the processing of pain (Lin et al., 2022). The use of a whole-brain ^1^H-MRSI technique in individuals with TBI (Govind et al., 2010; Maudsley et al., 2015; Lin et al., 2022) has largely overcome the spatial coverage limitation of the brain and permits the evaluation of metabolite alterations following TBI during acute and subacute phases (Govind et al., 2010; Harris et al., 2012; DeVience et al., 2017; Bartnik-Olson et al., 2021). Yet, there remains limited research investigating changes in NAA, tCho, Glx, tCre, and *m*-Ins among those reporting pain in the chronic disease stage.

Our laboratory has previously assessed the concentration of metabolite levels and their respective ratios among those with TBI and subacute pain (Widerström-Noga et al., 2016). However, no studies have examined such alterations in those with chronic pain following TBI. Furthermore, there is limited prior research that evaluated brain metabolite differences among individuals after TBI with- and without pain and pain-free healthy controls. Such information may provide insight into potential mechanisms associated with the development of chronic pain in those with TBI. Therefore, the purpose of this study was to obtain whole-brain ^1^H-MRSI measures in participants with chronic TBI and pain, and compare their metabolite profiles to those without pain and healthy controls across several pain-relevant brain regions. It was hypothesized that participants with chronic TBI and comorbid pain would exhibit altered brain metabolism compared to those with TBI and no pain and healthy controls. We also examined associations between metabolite levels, chronic pain symptoms, and psychological measures among the TBI with pain group.

## 2. Materials and methods

### 2.1. Study participants

Sixty-eight adult participants were enrolled in this study. Of them, thirty-one were healthy controls (HC, *N* = 31), and thirty-seven had closed-head TBI (TBI, *N* = 37). Participants with TBI were categorized into those with chronic pain (TBI-P, *N* = 19) and those without pain (TBI-NP, *N* = 18). Demographic and injury characteristics for all participants are shown in Table 1. In addition, all participants were: (1) fluent in English, (2) free from significant cognitive impairment using the MMSE-2 (PAR, 2020), (3) with no recent history of alcohol or drug abuse, (4) with no severe major depression, and (5) free from other neurological diseases (e.g., multiple sclerosis) or trauma (e.g., spinal cord injury). Enrolled TBI participants had experienced their injury six to two hundred seventy-six months before the start of the study and were thus considered to be in the chronic time period following injury (Mayer et al., 2017). The severity of TBI in each participant was determined based on the Glasgow Coma Scale (GCS) score when available. Participants were recruited through advertisements posted at the University of Miami Medical Campus and the Health and Human Services/National Institute on Disability, Independent Living, and Rehabilitation Research (HHS/NIDILRR) organization, South Florida TBI Model System center, the Transforming Research and Clinical Knowledge in Traumatic Brain Injury (TRACK-TBI) study center within the University of Miami (UM), and by word of mouth. TBI participants provided medical records with proof of head/brain injury obtained from their medical care provider or insurance company unless they were directly referred from the HHS/NIDILRR, South Florida TBI Model System center, or the TRACK-TBI study center. The institutional review board of the University of Miami approved the study protocol, and all participants signed an informed consent form before participation. The data presented in this article is a subset of a more extensive study involving pain, quantitative sensory and psychological/psychosocial evaluations, and brain imaging in individuals with TBI. Pain, quantitative sensory, and psychological/psychosocial data of this larger cohort have been published (Robayo et al., 2022).

**TABLE 1:** Demographic and injury characteristics for HC (*N* = 31), TBI-NP (*N* = 17), and TBI-P (*N* = 16) groups.

Variable	HC mean (SD)	TBI-NP mean (SD)	TBI-P mean (SD)	*p*
Gender (n)				0.870^a^
Male	15	9	9	
Female	16	8	7	
Age (years)	27.71 (7.72)	28.53 (8.16)	35.19 (10.77)	0.040^b^
MMSE-2	28.65 (1.31)	28.47 (2.12)	27.81 (1.94)	0.330^b^
Years of education	15.32 (3.11)	15.94 (2.73)	13.88 (1.78)	0.079^c^
Age at TBI (years)	-	21.88 (8.43)	31.50 (11.34)	0.006^c^
Time after TBI (months)	-	79.47 (74.17)	43.97 (26.88)	0.233^c^
GCS*	-	8.13 (4.94)	10.57 (4.69)	0.483^c^
Cause of injury (n) MVA Other	- -	12 5	8 8	0.279^d^

MVA, motor vehicle accident; MMSE-2, Mini-Mental State Examination – Version 2.

*GCS scores were available for 15 TBI participants.

^a^Chi-square, ^b^Kruskall-Wallis, ^c^Mann-Whitney U, ^d^Fisher’s exact test.

Pairwise comparisons of groups showed that age significantly differed between the TBI-P and HC groups (*p* = 0.040).

### 2.2. Proton-magnetic resonance spectroscopic imaging (^1^H-MRSI) data

#### 2.2.1. Data acquisition

Magnetic resonance spectroscopic imaging data from all participants (*N* = 68) were acquired using a 3 Tesla MRI scanner (Siemens Skyra) with a 20-channel receive-only head coil. The protocol included T_1_-weighted MPRAGE (magnetization-prepared acquisition rapid gradient echo; 1-mm isotropic resolution), T2-weighted gradient echo, FLAIR (fluid-attenuated inversion recovery), and whole-brain MR spectroscopic imaging (axial orientation, TE = 17.6 ms, TR = 1551 ms, field of view: 280 × 280 × 180 mm^3^, slab thickness: 135 mm, matrix: 50 × 50 × 18, nominal voxel volume: 0.31 cm^3^, and ∼ 17 min acquisition time).

#### 2.2.2. Spectral processing

RS data were processed using the MIDAS package (Maudsley et al., 2006) to obtain signal-normalized (institutional units), and CSF partial volume corrected metabolite maps at an approximate spatial volume of 1 cm^3^. Metabolite concentrations were obtained for NAA, tCre, tCho, Glx, and *m*-Ins. Individual subject T1-weighted MRI data were spatially transformed into a T1-weighted MRI in the Montreal Neurological Institute (MNI) template space. The resulting spatial transformation was subsequently applied to the MRSI data. We used a modified version (47 regions only, see Supplementary Table 1) of the anatomic parcellation-based Automated Anatomical Labeling (AAL) atlas (Tzourio-Mazoyer et al., 2002) in the MNI template space for obtaining metabolite data for each respective brain region. The quality of MRS data used for analysis was controlled by applying criteria such as linewidth (≤ 12 Hz), tissue fraction (> 70% gray matter plus white matter), and outlier removal (> 3 SD). To improve the quality of MRS data obtained for analysis, spectra from multiple voxels within a selected brain region were summed to create a spectrum (e.g., thalamus) using the Map INTegrated spectrum (MINT) tool in the MIDAS package (Maudsley et al., 2006). In addition, metabolite ratios (NAA/tCre, *m*-Ins/tCre, Cho/tCre, Glx/tCre, and Glx/*m*-Ins) were calculated by dividing corresponding metabolite values per area. Data from four TBI participants were excluded from the final analysis due to motion artifacts, insufficient water signal suppression, and significant signal distortions.

#### 2.2.3. Regions of interest

**FIGURE 1:**
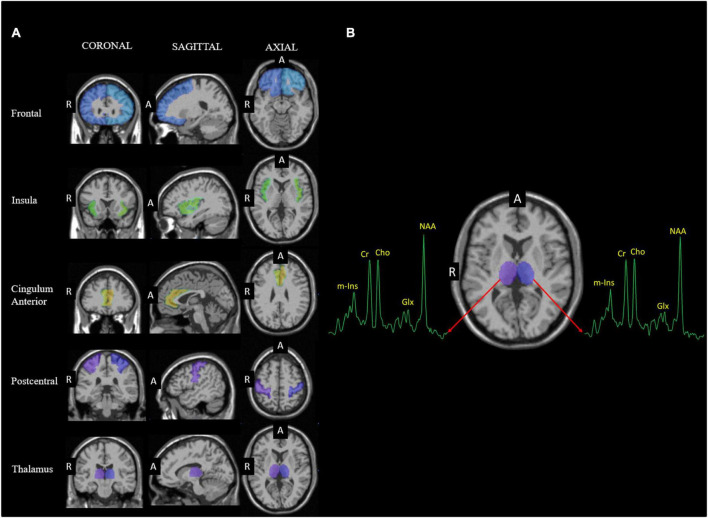
**(A)** Coronal, sagittal and axial slices showing the regions of interest (ROIs), including the frontal, cingulum anterior, insula, postcentral, and thalamus from left and right hemispheres, superimposed on template T1 MRI and obtained using the AAL atlas (63). **(B)** Representative spectrum from the thalamus. Abbreviations used for anterior (A) and right (R).

Data from ten pain-related brain regions of interest were selected for analysis. These included the left and right frontal, insula, cingulum anterior, postcentral, and thalamus (see Figure 1A), which are involved in the processing of sensory-discriminative (e.g., location and intensity), cognitive-evaluative, and affective-emotional aspects of nociception and pain (Bushnell et al., 2013; Kuner and Kuner, 2021; Mercer Lindsay et al., 2021). These ROIs were selected because metabolite alterations in these regions have been consistently observed in chronic pain conditions (Ito et al., 2017; Levins et al., 2019; Peek et al., 2020; Cruz-Almeida and Porges, 2021), including neuropathic pain (Chang et al., 2013; Widerström-Noga et al., 2015). The frontal region, which includes the prefrontal cortex, has been implicated in attentional and evaluative processes of pain (Bushnell et al., 2013; Kuner and Kuner, 2021; Tan and Kuner, 2021). The insula has been implicated in coding pain intensity (Garcia-Larrea et al., 2010; Garcia-Larrea and Peyron, 2013). The anterior cingulate cortex (ACC) and cingulum are components of the limbic system, thought to be involved in the affective-emotional components of the pain experience (Bushnell et al., 2013; Kuner and Kuner, 2021; Tan and Kuner, 2021). The postcentral gyrus or primary somatosensory cortex is commonly activated during induced pain and implicated in the sensory-discriminative (e.g., location and intensity) components of pain (Apkarian et al., 2005; Kuner and Kuner, 2021; Tan and Kuner, 2021). Lastly, the thalamus is the primary relay station for sensory information, communicates with the cortex and is also be involved in pain modulation (Apkarian et al., 2005).

### 2.3. Clinical variables

Pain and psychological function measures were obtained by interview using validated questionnaires.

#### 2.3.1. Pain

The pain severity subscale of the multidimensional pain inventory (MPI) (Kerns et al., 1985) was used to assess overall pain severity on a 0-6 scale. In addition, neuropathic pain symptoms were evaluated using the NPSI (Bouhassira et al., 2004). Total NPSI scores were obtained by summing all the individual scores for ten questions assessing the presence and severity of (1) spontaneous pain with burning, squeezing, or pressure characteristics; (2) painful attacks with electric shocks or stabbing characteristics; (3) pain provoked or increased by brushing, pressure, or contact with something cold on the painful area; and (4) abnormal sensations including pins and needles, and tingling.

#### 2.3.2. Psychological function

The Beck anxiety inventory (BAI) (Beck et al., 1988) and Beck depression inventory (BDI) (Steer et al., 2001) were used to evaluate the presence and severity of 21 anxiety and depression symptoms, respectively. Participants were asked to rate their symptoms over the past two weeks using a number ranging from 0 to 3, with increasing scores reflecting greater symptomatology. Similarly, the post-traumatic stress disorder (PTSD) checklist-civilian version, PCL-C (Weathers, 1993; Ruggiero et al., 2003; Haarbauer-Krupa et al., 2017), was used to evaluate the presence and severity of PTSD symptoms. The Rivermead Post-concussion Symptoms Questionnaire Rivermead was also used to assess awareness of cognitive, emotional, and physical symptoms following TBI (King et al., 1995).

### 2.4. Statistical analysis

Independent variables were assessed for normality and homogeneity of variances (homoscedasticity) using Shapiro-Wilk and Levene’s tests, respectively. Mean values for each of the metabolites (NAA, *m*-Ins, tCho, Glx and tCre) were compared across the three groups (HC versus TBI-NP and TBI-P) using multiple non-parametric ANCOVAs (Quade, 1967) with age as a covariate. LSD pairwise comparisons were obtained. In addition, metabolite ratios (NAA/tCre, *m*-Ins/tCre, Cho/tCre, Glx/tCre, and Glx/*m*-Ins) were compared using Kruskal-Wallis Tests. Statistical analyses were conducted using SPSS. Results were considered significant if values were *p* ≤ 0.05 after being corrected for multiple comparisons using the Benjamini and Hochberg false discovery rate (FDR) method (Benjamini and Hochberg, 1995). Data from the frontal, anterior cingulum, insula, postcentral gyrus, and thalamic regions were analyzed separately for both the left and right hemispheres. To evaluate the associations between clinical variables (e.g., pain and psychological) and the metabolite measures in the TBI-P group, we conducted partial Pearson correlations with age as a covariate.

## 3. Results

### 3.1. Pain and psychological characteristics of participants

Pain and psychological characteristics are indicated in Table 2.

**TABLE 2:** Pain and psychological characteristics for the HC (*N* = 31), TBI-NP (*N* = 17), and TBI-P (*N* = 16) groups.

Variable	HC mean (SD)	TBI-NP mean (SD)	TBI-P mean (SD)	*p*	Pairwise comparisons		
					HC vs. TBI-P	TBI-NP vs. TBI-P	HC vs. TBI-NP
Total NPSI	–	–	37.38 (29.29)	–	–	–	–
Pain severity	–	–	3.98 (1.37)	–	–	–	–
Total BAI	5.39 (7.92)	5.23 (6.06)	21.69 (11.86)	<0.001^a^	<0.001	0.002	ns
Total BDI	4.52 (5.45)	6.76 (7.19)	18.50 (10.24)	<0.001^a^	<0.001	0.008	ns
Total PCL-C	25.65 (10.20)	24.76 (10.34)	41.56 (16.58)	0.002^a^	0.004	0.008	ns
Total Rivermead	–	14.06 (12.34)	36.69 (16.17)	<0.001^b^	–	–	–

NPSI, neuropathic pain symptom inventory; BDI, Beck depression inventory; BAI, Beck anxiety inventory, PCL-C, Post-traumatic stress disorder checklist – civilian version; Rivermead, Rivermead post-concussion symptoms questionnaire.

^a^Kruskall-Wallis test; ^b^Mann-Whitney U test.

Pairwise comparisons were adjusted by the Bonferroni correction for multiple comparisons. ns, not significant.

### 3.2. Metabolite differences between the HC, TBI-NP and TBI-P groups

**FIGURE 2:**
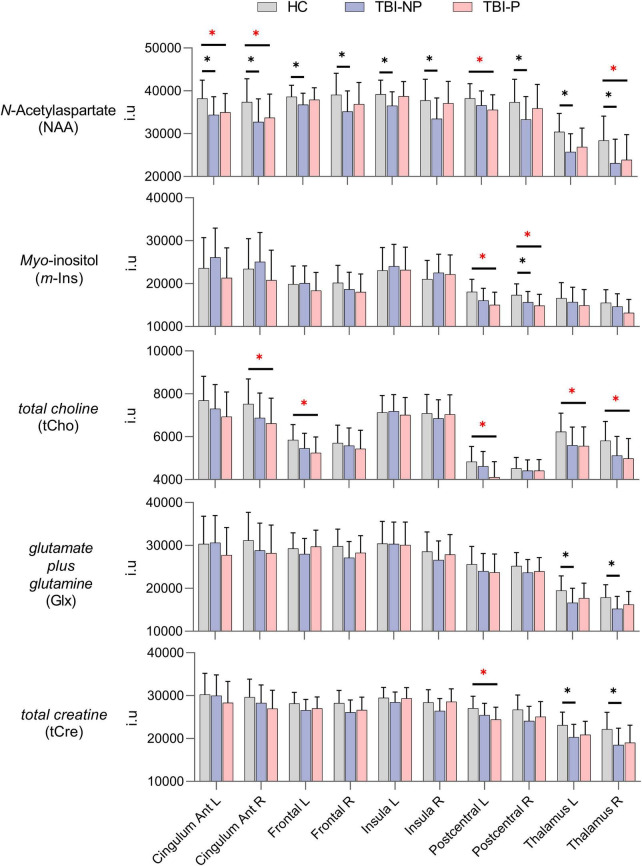
Mean values of the metabolites in the healthy control (HC, *N* = 31), traumatic brain injury with no chronic pain (TBI-NP, *N* = 17), and traumatic brain injury with chronic pain (TBI-P, *N* = 16) groups, by brain region, with error bars indicating the standard deviation (SD). False discovery rate (FDR)-corrected significant differences indicated with asterisks (*p ≤ 0.05). Red asterisks (*) indicate significant differences between TBI-P and other groups. The *y*-axis scale is in institutional units (i.u.). Abbreviations used for the hemispheric regions: R, right and L, left; Ant, anterior.

Results from non-parametric ANCOVA are presented in Tables 3-7, with Figure 2 displaying age-corrected means and pairwise comparisons for each metabolite (i.e., NAA, tCho, *m*-Ins, Glx, and tCre) in the selected ROIs for each group. Relative to HC, those within the TBI-NP group showed significantly lower NAA in nearly all brain regions, excluding the left postcentral ROI. These participants also displayed significantly lower *m*-Ins within the right postcentral region and significantly lower Glx and tCre within the right and left thalamus. When those in the TBI-P group were compared to HC, significantly lower NAA was also observed in the left and right anterior cingulum, left postcentral region, and the right thalamus. Similarly, tCho was significantly lower in the left frontal, left postcentral, right cingulum, and left and right thalamus, with tCre showing significantly lower values in the left postcentral region.

**TABLE 3:** Results from analysis of covariance (ANCOVA) controlling for age between HC (*N* = 31), TBI-NP (*N* = 17) and TBI-P (*N* = 16) groups for *N*-acetylaspartate (NAA).

ROI	HC mean^#^ (SD)	TBI-NP mean^#^ (SD)	TBI-P mean^#^ (SD)	p*	Pairwise comparisons		
					HC vs. TBI-NP	HC vs. TBI-P	TBI-P vs. TBI-NP
Cingulum Ant L	37,284 (7,196)	34,443 (4,337)	33,553 (6,248)	0.035	0.022	0.042	0.936
Cingulum Ant R	36,662 (6,176)	32,826 (8,338)	31,932 (6,828)	0.023	0.016	0.045	0.995
Frontal L	39,082 (3,777)	37,065 (2,522)	36,453 (3,038)	0.044	0.020	0.233	1.073
Frontal R	38,835 (4,327)	35,329 (8,589)	35,834 (2,647)	0.023	0.018	0.110	1.006
Insula L	39,552 (3,166)	36,741 (2,667)	37,600 (4,724)	0.024	0.020	0.633	0.310
Insula R	38,125 (3,692)	33,687 (7,196)	35,693 (4,852)	0.040	0.013	0.593	0.375
Postcentral L	38,681 (3,987)	36,874 (2,846)	34,588 (4,038)	0.044	0.095	0.050	0.760
Postcentral R	37,802 (3,556)	33,610 (8,735)	34,355 (4,756)	0.046	0.018	0.223	0.910
Thalamus L	30,758 (3,690)	25,940 94,421)	25,293 (6,182)	0.010	0.010	0.065	0.806
Thalamus R	28,637 (3,894)	23,221 (7,099)	22,462 (7,417)	0.030	0.013	0.040	0.886

^#^Values are in institutional units (i.u). Abbreviations used for the hemispheric regions: R, right and L; left, Ant, anterior.

*p-values were corrected for multiple testing using the false discovery rate (FDR) method.

**TABLE 4:** Results from analysis of covariance (ANCOVA) controlling for age between HC (*N* = 31), TBI-NP (*N* = 17) and TBI-P (*N* = 16) groups for *myo-i*nositol (*m*-Ins).

ROI	HC mean^#^ (SD)	TBI-NP mean^#^ (SD)	TBI-P mean^#^ (SD)	*p* *	Pairwise comparisons		
					HC vs. TBI-NP	HC vs. TBI-P	TBI-P vs. TBI-NP
Cingulum Ant L	22,807 (7,207)	26,109 (10,080)	23,494 (10,272)	0.520	0.778	0.408	1.580
Cingulum Ant R	24,123 (8,210)	25,082 (9,461)	21,281 (7,032)	0.416	0.708	0.203	1.135
Frontal L	22,806 (12,705)	20,198 (4,216)	18,344 (3,286)	0.584	0.831	0.371	0.887
Frontal R	20,323 (4,174)	18,732 (3,963)	18,602 (5,192)	0.465	0.387	0.302	1.158
Insula L	28,640 (22,080)	24,257 (6,517)	22,437 (4,596)	0.863	0.769	0.630	1.038
Insula R	21,124 (3,697)	22,582 (4,216)	23,270 (7,306)	0.600	0.508	0.369	1.223
Postcentral L	18,687 (3,879)	16,195 (2,594)	15,101 (3,325)	0.015	0.110	0.010	0.843
Postcentral R	17,741 (2,648)	15,705 (2,877)	14,933 (3,026)	0.020	0.030	0.020	0.995
Thalamus L	17,258 (4,796)	15,714 (3,126)	14,671 (3,377)	0.506	0.787	0.305	1.010
Thalamus R	15,697 (3,326)	14,576 (2,949)	14,281 (4,382)	0.310	0.620	0.103	0.973

^#^Values are in institutional units (i.u). Abbreviations used for the hemispheric regions: R, right and L, left; Ant, anterior.

*p-values were corrected for multiple testing using the false discovery rate (FDR) method.

**TABLE 5:** Results from analysis of covariance (ANCOVA) controlling for age between HC (*N* = 31), TBI-NP (*N* = 17) and TBI-P (*N* = 16) groups for total choline (tCho).

ROI	HC mean^#^ (SD)	TBI-NP mean^#^ (SD)	TBI-P mean^#^ (SD)	*p* *	Pairwise comparisons		
					HC vs. TBI-NP	HC vs. TBI-P	TBI-P vs. TBI-NP
Cingulum Ant L	7,452 (1,725)	7,299 (1,061)	6,841 (938)	0.313	0.780	0.115	1.110
Cingulum Ant R	7,314 (1,417)	6,873 (1,615)	6,658 (743)	0.148	0.690	0.050	1.485
Frontal L	5,868 (842)	5,469 (444)	5,197 (584)	0.078	0.293	0.043	0.906
Frontal R	5,738 (895)	5,603 (691)	5,371 (795)	0.396	0.781	0.159	0.923
Insula L	7,152 (782)	7,200 (716)	6,926 (808)	0.970	0.780	0.756	0.807
Insula R	7,120 (886)	6,870 (911)	6,928 (789)	0.939	0.669	0.760	1.166
Postcentral L	4,864 (797)	4,635 (650)	4,047 (464)	0.090	0.752	0.020	0.460
Postcentral R	4,624 (601)	4,443 (578)	4,303 (482)	0.856	0.738	0.704	0.964
Thalamus L	6,253 (835)	5,608 (879)	5,450 (862)	0.050	0.150	0.048	1.063
Thalamus R	5,815 (833)	5,126 (952)	4,817 (1,107)	0.055	0.170	0.030	0.750

^#^Values are in institutional units (i.u). Abbreviations used for the hemispheric regions: R, right and L, left; Ant, anterior.

*p-values were corrected for multiple testing using the false discovery rate (FDR) method.

**TABLE 6:** Results from analysis of covariance (ANCOVA) controlling for age between HC (*N* = 31), TBI-NP (*N* = 17) and TBI-P (*N* = 16) groups for glutamate and glutamine (Glx).

ROI	HC mean^#^ (SD)	TBI-NP mean^#^ (SD)	TBI-P mean^#^ (SD)	*p* *	Pairwise comparisons		
					HC vs. TBI-NP	HC vs. TBI-P	TBI-P vs. TBI-NP
Cingulum Ant L	30,757 (10,229)	30,673 (6,776)	27,734 (5,001)	0.549	0.970	0.498	1.245
Cingulum Ant R	32,792 (13,348)	28,918 (8,292)	30,015 (11,732)	0.531	0.601	0.307	1.001
Frontal L	29,645 (3,870)	28,222 (3,911)	29,233 (4,000)	0.831	0.684	0.914	1.180
Frontal R	30,118 (5,136)	27,269 (5,480)	27,627 (27,412)	0.303	0.140	0.725	1.242
Insula L	30,651 (4,980)	30,467 (5,882)	29,244 (4,600)	0.964	0.877	0.926	0.979
Insula R	29,362 (4,522)	26,752 (5,579)	27,095 (4,771)	0.568	0.437	0.406	1.096
Postcentral L	25,982 (4,760)	24,221 (4,005)	22,809 (3,549)	0.604	0.424	0.657	0.952
Postcentral R	25,464 (2,793)	23,816 (3,339)	23,477 (3,738)	0.578	0.260	0.360	1.053
Thalamus L	19,644 (3,871)	16,735 (2,232)	18,974 (7,859)	0.160	0.050	0.342	2.430
Thalamus R	18,546 (3,710)	15,351 (2,056)	16,059 (3,933)	0.030	0.010	0.250	1.143

^#^Values are in institutional units (i.u). Abbreviations used for the hemispheric regions: R, right and L, left; Ant, anterior.

*p-values were corrected for multiple testing using the false discovery rate (FDR) method.

**TABLE 7:** Results from analysis of covariance (ANCOVA) controlling for age between HC (*N* = 31), TBI-NP (*N* = 17) and TBI-P (*N* = 16) groups for total creatine (tCre).

ROI	HC mean^#^ (SD)	TBI-NP mean^#^ (SD)	TBI-P mean^#^ (SD)	*p* *	Pairwise comparisons		
					HC vs. TBI-NP	HC vs. TBI-P	TBI-P vs. TBI-NP
Cingulum Ant L	29,329 (5,892)	29,946 (7,433)	27,679 (4,589)	0.087	0.065	0.147	1.063
Cingulum Ant R	28,812 (5,011)	28,273 (6,272)	26,496 (3,973)	0.110	0.131	0.082	0.876
Frontal L	28,239 (3,120)	26,622 (1,648)	26,809 (1,833)	0.084	0.057	0.141	0.972
Frontal R	27,800 (3,337)	26,109 (4,099)	26,636 (1,878)	0.118	0.101	0.134	1.037
Insula L	29,583 (2,439)	28,505 (1,322)	29,056 (2,991)	0.253	0.127	0.945	0.553
Insula R	28,838 (2,821)	26,469 (4,036)	28,176 (2,765)	0.111	0.070	1.038	0.660
Postcentral L	27,194 (3,165)	25,518 (2,440)	24,149 (2,208)	0.025	0.123	0.010	0.620
Postcentral R	26,805 (2,489)	24,166 (5,159)	24,738 (2,352)	0.088	0.064	0.083	0.981
Thalamus L	23,211 (2,801)	20,390 (2,486)	20,264 (3,928)	0.030	0.020	0.090	1.193
Thalamus R	22,252 (3,327)	18,548 (4,116)	18,219 (5,066)	0.027	0.025	0.077	0.937

^#^Values are in institutional units (i.u). Abbreviations used for the hemispheric regions: R, right and L, left; Ant, anterior.

*p-values were corrected for multiple testing using the false discovery rate (FDR) method.

As indicated in Table 1, “time after TBI” is not significantly different between TBI groups (TBI-NP vs. TBI-P). However, we understand that this variable covers a large range. For that, we conducted a sub-analysis to test if “time after TBI” affect the between-group results. Independent-Samples Mann-Whitney U tests, between a short time after TBI (N = 17, mean = 24.15) vs. a long time after TBI (N = 16, mean = 102.75) groups, indicated that there was no effect of “time after TBI” on metabolite levels, except for *m*-Ins on the postcentral right region (U = 71.00, p = 0.02). Myo-Inositol levels were significantly higher in the group with a shorter time after TBI (mean rank = 20.82) vs. a longer time after TBI (mean rank = 12.94). We then conducted an additional non-parametric ANCOVA for myo-inositol in the postcentral right region, controlling for “age” and “time after TBI.” Similar to our previously reported results, there was no significant difference between TBI-P vs. TBI-NP groups, F (1, 31) = 0.278, *p* = 0.602, suggesting that even though “time after TBI” may affect myo-inositol levels, the effect was not strong.

Comparisons among the groups of NAA/tCre, Cho/tCre, Glx/tCre, *m*-Ins/tCre, and Glx/*m*-Ins ratios did not indicate significant differences between TBI-NP and TBI-P relative to controls (data not shown), except for higher *m*-Ins/Cre and lower Glx/*m*-Ins ratios in the right insula. Although uncorrected *p*-values were significant (*p* = 0.034 and *p* = 0.018), they did not survive FDR correction for multiple tests.

### 3.3. Associations with pain, psychological and injury variables

Correlation coefficients between metabolite levels and clinical variables for the TBI-P group are provided in Table 8. Age was used as a covariate in the analysis to control for any potential confounding effect that age may have on metabolite measures. Significant positive correlations were found between 1) left frontal NAA levels and total NPSI scores, BDI and PTSD symptoms, and Rivermead scores, and 2) right frontal NAA levels with pain severity and Rivermead scores. Additionally, left postcentral NAA values were significantly correlated to total NPSI scores, suggesting some relationship between the severity of neuropathic pain symptoms and altered NAA levels.

**TABLE 8:** Pearson correlation coefficients (r) of partial correlations between *N*-acetylaspartate (NAA) levels of the TBI pain group (TBI-P, *N* = 16) and pain and psychological variables, controlling for age.

*N*-acetylaspartate (NAA)	Pain variables		Psychological variables			
	Total NPSI	Pain Severity	BDI	BAI	PCL-C	Rivermead
	r values					
Cingulum Ant L	−0.012	0.177	−0.133	−0.246	−0.245	0.026
Cingulum Ant R	0.323	**0.479**	0.162	0.219	0.096	0.367
Frontal L	0.599*	**0.511**	0.589*	**0.485**	0.535*	0.556*
Frontal R	**0.519**	0.524*	**0.414**	**0.470**	0.325	0.577*
Insula L	**0.444**	0.386	0.192	0.062	0.011	0.351
Insula R	0.326	0.198	−0.172	−0.005	−0.289	0.151
Postcentral L	0.555*	**0.442**	0.303	0.342	**0.419**	0.367
Postcentral R	0.299	0.138	0.037	0.068	0.058	0.38
Thalamus L	−0.005	−0.196	−0.076	−0.344	−0.165	0.165
Thalamus R	0.243	0.116	−0.086	−0.099	−0.177	0.286

Age was used as a covariate. Asterisks indicate significant correlations (*p ≤ 0.05), uncorrected for multiple comparisons. Abbreviations are used for the right (R) and left (L) hemispheric regions. BDI, Beck depression inventory; BAI, Beck anxiety inventory; PCL-C, post-traumatic stress disorder checklist – civilian version, Rivermead, Rivermead post-concussion symptoms questionnaire. Moderate to strong correlations (*r* ≥ 0.4) are indicated in bold.

## 4. Discussion

Whole-brain ^1^H-MRSI can provide *in vivo* brain tissue metabolite levels following TBI (Govindaraju et al., 2000; Govind et al., 2010). This type of information can help elucidate the relationship between brain tissue metabolite alterations in people with chronic TBI and co-occurring chronic pain. In this study, we compared NAA, tCho, Glx, tCre, and *m*-Ins levels and their respective ratios across frontal, cingulate, insula, somatosensory, and thalamic regions in participants living with chronic TBI and healthy controls. We also examined the relationships between metabolite levels and pain and psychological measures in those with chronic pain following TBI. Relative to controls, those in the TBI and chronic pain group exhibited significantly lower NAA in the left and right anterior cingulum, left postcentral region, and the right thalamus. Similarly, tCho was significantly lower in the left frontal, left postcentral, right cingulum, and left and right thalamus, with tCre showing significantly lower values in the left postcentral region. Conversely, those with TBI without chronic pain also showed significantly lower NAA in nearly all selected ROIs, excluding the left postcentral. These participants also displayed significantly lower *m*-Ins within the right postcentral region and significantly less Glx and tCre within the right and left thalamus. In addition, we observed significant correlations between left frontal NAA levels and poorer BDI scores, worse PTSD symptoms, and higher post-concussive symptoms among those reporting chronic pain. Similarly, left frontal NAA levels were correlated to pain severity and post-concussive symptoms, while left postcentral NAA was correlated with total NPSI scores. These findings may indicate long-term alterations among specific metabolite levels, such as NAA, that could be used as biomarkers for psychological dysfunction and chronic pain, including neuropathic pain symptoms, following TBI.

Although cross-sectional and longitudinal studies have identified acute and subacute reductions in NAA or NAA/Cr as clinical indicators of neurological dysfunction, some report recovery to values approximating healthy controls (Govind et al., 2010; Maudsley et al., 2015, 2017). In general, lower NAA levels or NAA/tCre ratios are associated with worse TBI outcomes (Govind et al., 2010; Croall et al., 2015; Holshouser et al., 2019) and with chronic neuropathic pain conditions (Pattany et al., 2002; Fukui et al., 2006; Sorensen et al., 2008). Our findings support these observations and reveal TBI-associated alterations in NAA levels across numerous ROIs relative to healthy controls. Lower NAA levels in participants with TBI during the chronic stage likely reflect a reduction in the metabolic integrity and overall viability of neurons housed across pain-related structures, as NAA is one of the most abundant and highly concentrated neuronal metabolites responsible for mitochondrial bioenergetics and the transfer of intermediary energy substrates to various glial cells, including oligodendrocytes (Govindaraju et al., 2000; Moffett et al., 2013). These findings align with those of Lin and colleagues (2022), who recently used whole-brain ^1^H-MRSI to examine metabolite differences in participants suffering from moderate to severe TBI at least 12 months after their initial injury. Although Lin and colleagues (2022) reported lower NAA and its ratios across several ROI, metabolite levels were not statistically different in the TBI group compared to healthy controls following FDR correction for multiple comparisons (Lin et al., 2022). Our study develops this line of inquiry further and confirms metabolite alterations in people with TBI during the chronic stage, with *p* values surviving multiple comparisons.

Biochemical and structural abnormalities among key pain regions, including the frontal cortex (Glodzik-Sobanska et al., 2007), insula (Widerström-Noga et al., 2016), the primary somatosensory cortex (Ferris et al., 2021), and thalamus (Gustin et al., 2014), have been identified following neurotrauma, suggesting an emergent schematic of disrupted metabolic and structural integrity, and reorganization of existing neural tissue. To our knowledge, no studies have delineated metabolite alterations in those with chronic pain following TBI. However, our findings may also indicate that brain tissue metabolite alterations following TBI are necessary but not sufficient for developing chronic pain. Thus, a combination of cellular and molecular mechanisms, including altered neuronal and glial cell function or loss within the pain circuitry, may lead to maladaptive plasticity favoring persistent pain symptoms post-TBI. Indeed, maladaptive plasticity has been associated with neuropathic pain (Costigan et al., 2009). Although not significantly different from the non-pain TBI group, apparent higher metabolite levels in the bilateral frontal and insular regions, right postcentral, and right thalamus were observed among those with chronic pain. This observation may indicate a shift toward increased metabolic activity (hyperactivity) or loss of inhibitory control associated with a state of central sensitization within pain-related brain regions, also supported by positive correlations between NAA levels in the left and right frontal and left postcentral regions and pain measures. Similarly, NAA levels in the left and right frontal regions were also positively correlated with measures of depression, post-traumatic stress disorder, and post-concussion symptoms. Neuroimaging studies suggest that abnormal frontal executive connectivity largely contributes to developing chronic pain symptoms where the intensity and temporality are often comprised of affective and motivational elements (Davis and Moayedi, 2013). Circuitry housed within frontal brain regions is thought to provide top-down attentional control of salient sensory information while also representing the emotional content of pain within more midline regions (Kragel et al., 2018).

The interpretation of tCho signal alterations within the selected ROIs is difficult to parse, mainly because the tCho signal includes contributions from free choline (Cho), glycerophosphorylcholine (GPC), and phosphorylcholine (PC) (Govindaraju et al., 2000). Cho is required for the synthesis of neuronal and glial membrane phospholipids (e.g., phosphatidylcholine) and the neurotransmitter acetylcholine (Ach), which is involved in sensory, motor, and cognitive function. GPC acts as a cerebral osmolyte, whereas PC is involved in the synthesis of myelin sheath-associated compounds (e.g., sphingomyelin), which is essential for axonal integrity (Govindaraju et al., 2000; Leipelt and Merrill, 2004; Javaid et al., 2021). Higher tCho levels have been consistently reported during the acute and subacute TBI stages, and this increase has been attributed to breakdown products of cell membranes and myelin and astrogliosis (Govind et al., 2010). Contrary to this general finding, both TBI groups showed lower tCho levels when compared to the healthy control group in the left thalamus. Moreover, those in the TBI and chronic pain group had lower tCho levels within the right cingulum, left frontal, left postcentral, and the right thalamic regions when compared to healthy controls. These reductions likely suggest permanent tissue damage and no further possibility of structural repairs, which manifest as a decreased tCho profile that differs from those in the acute and subacute injury stages. Future research with larger sample sizes is required to confirm this finding. Ultimately, our results showing lower tCho in the cingulum anterior, frontal, postcentral, and thalamic regions in the TBI and chronic pain group relative to the healthy control group may suggest either one or a combination of the following: (1) decreased membrane turnover or impaired cholinergic neurotransmission (i.e., Cho), (2) reduced cell volumes (i.e., GPC), or (3) decreased myelin synthesis (i.e., PC) in the chronic stage.

As with tCho levels, the impact of lower *m*-Ins is difficult to delineate, given limited findings across previously published MRS studies (Kierans et al., 2014). Indeed, prior evidence suggests that *m*-Ins is a crucial marker for glial cell health and also serves as an osmolyte – with higher levels reflecting greater neuronal dysfunction through alterations in calcium signaling and other ion mediated transduction pathways (Fisher et al., 2002; Croall et al., 2015). Similar to tCho, studies in acute and subacute TBI generally report elevated *m*-Ins levels or its ratios (Garnett et al., 2000a; Kierans et al., 2014). Our results indicate that those with TBI and chronic pain presented with lower *m*-Ins levels across the left postcentral, and both the right and left thalamus regions when compared to healthy controls. This result differs from those reported by Yoon and colleagues (Yoon et al., 2005), who observed elevated *m*-Ins/Cre ratios among those with moderate to severe TBI five-months after their injury. Once again, reduced neurometabolite levels may suggest tissue damage and metabolic dysfunction within neural networks responsible for both the gating of sensory information (i.e., thalamus) and processing of such information within higher order brain centers (i.e., left postcentral region). Additional studies in long term TBI are required to replicate such findings.

TBI-associated increases in tCre (creatine/phosphocreatine) have been documented with limited evidence suggesting that increased levels may reflect a combination of neuronal cell death paired with low grade gliosis (Garnett et al., 2001). While authors have noted altered tCre and its ratios in the acute (Gasparovic et al., 2009) and chronic TBI stages (Cadoux-Hudson et al., 1990), many assume constant levels of this metabolite when acquiring such measures, making observable variations difficult to contextualize across studies. Indeed, one study found no change in overall tCre levels in seven acute TBI cases (Garnett et al., 2001), while another observed increases in the neurometabolite that also correlated to more severe emotional distress and executive function performance in those with mild injuries (Gasparovic et al., 2009). That said, tCre represents an intracellular estimate of energy production, and when combined with the quantification of adenosine di/triphosphate (ADP and ATP), and inorganic phosphate (Pi), may provide a high-resolution marker for the metabolism of discrete brain regions associated with the processing of chronic pain symptoms. Based on the current findings and similar to those observed for *m*-Ins and lower tCre levels in the postcentral region of the TBI with chronic pain group relative to the healthy control group may suggest reduced glial cell density or volume (i.e., *m*-Ins) and reduced cellular energetics (i.e., tCre) (Govindaraju et al., 2000; Croall et al., 2015) within the left somatosensory cortex. Future studies are needed in larger cohorts to better examine variations in tCre years after TBI.

Increases in Glx levels within gray and white matter have been associated with poor neurological outcomes in individuals with TBI (Ashwal et al., 2004; Shutter et al., 2004), possibly reflecting excitotoxicity. However, lower Glx levels have also been detected in individuals with TBI compared to controls (Kubas et al., 2010; Yeo et al., 2011). Such findings may indicate downregulation of glutamate or glutamine production as a neuroprotective adaptation to neuronal death (Ramonet et al., 2004) or an overall reduced neuronal activity (Yeo et al., 2011). Based on a recent meta-analysis (Joyce et al., 2022), differences in findings may be attributed to biologically relevant (e.g., brain region) and technical factors (e.g., data acquisition). Results from Joyce et al. also showed that Glx was commonly unaffected, similar to our results, where no significant difference was found among groups with respect to all regions of interest except for the thalamus.

Ultimately, the global perception of pain is contingent on the proper integration of excitatory and inhibitory processes across brain networks, where functional and structural connectivity within these networks is essential for processing sensory-discriminative, affective-motivational, and cognitive-evaluative dimensions of pain (Treede et al., 2000; Apkarian et al., 2005; Kuner and Kuner, 2021; Mercer Lindsay et al., 2021; Tan and Kuner, 2021). Exposure of cortical and subcortical tissue to external forces of an appropriate magnitude may cause diffuse axonal injury and brain swelling (Werner and Engelhard, 2007). Coincidently, secondary injury pathologies such as neuroinflammation, abnormal monoaminergic signaling, and mitochondrial dysfunction may prompt a maladaptive reorganization of existing pain circuitry (Irvine and Clark, 2018) following TBI. This notion is further corroborated by parallel cell signaling pathologies that have been observed in those with spinal cord injury (SCI), painful diabetic neuropathy, trigeminal neuralgia, and complex regional pain syndrome (CRPS) (Chang et al., 2013).

### 4.1. Limitations

The associations between metabolite levels and severity of initial TBI were not investigated in this study but have been reported previously by us and others in cross-sectional and longitudinal studies (Garnett et al., 2000a,b; Govind et al., 2010), showing conflicting results. Thus, associations between metabolites in the context of initial TBI severity remain to be fully elucidated, as well as any influence of the mechanism of injury (e.g., MVA vs. sports injury), pain medication, and damage to the brain tissue microstructure. In addition, the relatively small sample size of the present study may have affected the statistical power when comparing both TBI groups (with and without chronic pain), so future studies should be conducted with larger sample sizes to further expand and validate the presented results. Furthermore, age and age at TBI were significantly different among groups. Participants in the TBI and chronic pain group were significantly older than those in the TBI without chronic pain group. For that reason, we controlled for age in our between-group comparisons to account for possible confounding effects of age on metabolite levels (Brooks J. C. W. et al., 2001; Grachev and Apkarian, 2001; Reyngoudt et al., 2012). Interestingly, age at TBI were higher in the TBI and chronic pain group than in the TBI without chronic pain group, suggesting that an older age at TBI may predispose people to chronic pain. This observation is consistent with previous literature reporting that an older age at TBI was related to greater disability (Forslund et al., 2019; Rabinowitz et al., 2021). However, any association between age at TBI and chronic pain remains to be fully elucidated, as well as the effects of age at TBI on long-term neurometabolite alterations.

## 5. Conclusion

The results of the present study suggest that those with chronic TBI present with significant neurometabolite alterations within pain-related brain regions. The extent of these alterations varied among the metabolite measured and the regions of interest. The presence of chronic pain was associated with significantly lower NAA, *m*-Ins, tCho and tCre levels across pain-related regions relative to controls, and with higher (although non-significant) NAA and tCre levels in the frontal, insula, right postcentral and thalamus regions compared to individuals without chronic pain. We also observed significant correlations between NAA levels with measures of pain and psychological factors, further emphasizing the relationship between the severity of pain and the extent of psychological distress among those with TBI. Altogether, these results may suggest that metabolite alterations are necessary but not sufficient for the maintenance of chronic pain post-injury and that additional underlying mechanisms, including maladaptive plasticity and functional reorganization in pain-related brain regions, may play a role in the development of chronic pain.

## Data availability statement

The datasets presented in this study can be found in online repositories. The names of the repository/repositories and accession number(s) can be found below: Home | FITBIR (nih.gov).

## Ethics statement

The studies involving human participants were reviewed and approved by University of Miami Institutional Review Board. The patients/participants provided their written informed consent to participate in this study.

## Author contributions

EW-N, VG, and AM designed the study. LR, VG, TS, and SS analyzed the proton-magnetic resonance spectroscopic imaging data. LR, EW-N, and NC analyzed the pain and psychological measures. NC, LR, and TS prepared the manuscript draft. All authors substantially contributed to the interpretation of data and manuscript revision.
