# Supplementary material for: Neurometabolite alterations in traumatic brain injury and associations with chronic pain
Source: Front Neurosci. 2023 Feb 23;17:1125128. doi: 10.3389/fnins.2023.1125128 (PMC9997848; doi:10.3389/fnins.2023.1125128)
Supplement: Supplementary file 1 [file Table_1.docx]

Supplementary Material

**Brain Metabolite Alterations in Chronic Traumatic Brain Injury and the Association with Chronic Pain**

| 1. Precentral R |
| --- |
| 1. Precentral L |
| 1. Frontal R |
| 1. Frontal L |
| 1. Rolandic Oper R |
| 1. Rolandic Oper L |
| 1. Supp Motor Area R |
| 1. Supp Motor Area L |
| 1. Insula R |
| 1. Insula L |
| 1. Cingulum Ant R |
| 1. Cingulum Ant L |
| 1. Cingulum Mid R |
| 1. Cingulum Mid L |
| 1. Cingulum Post R |
| 1. Cingulum Post L |
| 1. Hippocampus R |
| 1. Hippocampus L |
| 1. Calcarine R |
| 1. Calcarine L |
| 1. Cuneus R |
| 1. Cuneus L |
| 1. Lingual R |
| 1. Lingual L |
| 1. Occipital R |
| 1. Occipital L |
| 1. Fusiform R |
| 1. Fusiform L |
| 1. Postcentral R |
| 1. Postcentral L |
| 1. Parietal R |
| 1. Parietal L |
| 1. Precuneus R |
| 1. Precuneus L |
| 1. Paracentral Lobule R |
| 1. Paracentral Lobule L |
| 1. Caudate R |
| 1. Caudate L |
| 1. Putamen R |
| 1. Putamen L |
| 1. Pallidum R |
| 1. Pallidum L |
| 1. Thalamus R |
| 1. Thalamus L |
| 1. Temporal R |
| 1. Temporal L |
| 1. Cerebellum |
